# Morphology of Cortical Microglia in the Hyperacute Phase of Subarachnoid Hemorrhage

**DOI:** 10.3390/biology13110917

**Published:** 2024-11-12

**Authors:** Maksim Lyubomudrov, Anastasiya Babkina, Zoya Tsokolaeva, Mikhail Yadgarov, Sergey Shigeev, Dmitriy Sundukov, Arkady Golubev

**Affiliations:** 1 Federal Research and Clinical Center of Intensive Care Medicine and Rehabilitology, Moscow 107031, Russia; 2 Bureau of Forensic Medical Examination of the Department of Healthcare of the City of Moscow, Moscow 115516, Russia; 3 Institute of Medicine, Peoples’ Friendship University of Russia Named after Patrice Lumumba, Moscow 117198, Russia

**Keywords:** microglia, stroke, subarachnoid hemorrhage

## Abstract

Hemorrhagic stroke is the deadliest type of stroke. Cellular and molecular biomarkers are important for understanding the pathophysiology of stroke. Microglia are among the most promising biological markers. However, the morphological and physiological characteristics of microglia, as well as the structural and functional aspects of their interactions with neurons and other cells, are largely unknown. Due to the large number of different morphological phenotypes and very limited information on microglial changes in subarachnoid hemorrhage (SAH), we performed this study aimed at identifying the features of the distribution of various microglial phenotypes in the layers of the cerebral cortex in the hyperacute phase of non-traumatic SAH. We studied the distribution of various microglial phenotypes in the layers of the cerebral cortex of SAH non-survivors with a control group (coronary heart disease and sudden cardiac death were the underlying causes of death). An immunohistochemical study using antibodies to iba-1 (a marker of microglia) revealed changes in the morphological phenotypes of microglia in the cerebral cortex after subarachnoid hemorrhage. Significant differences between the groups indicate a rapid microglial response to injury. The findings indicate that there are quantitative and phenotypic changes in microglia in the cerebral cortex during early SAH in the human cortex.

## Simple Summary

Stroke is the one of leading causes of death worldwide. Hemorrhagic stroke is the deadliest type of stroke. Cellular and molecular biomarkers are important for understanding the pathophysiology of stroke. Microglia are among the most promising biological markers. Microglia cells perform many functions such as maintaining intercellular space by engulfing debris, scanning the microenvironment, and reacting to damage, and it can also show both regenerative and phagocytic activity. The multifunctionality of microglia determines the diversity of its phenotypes. The aim of our study was to evaluate the distribution of microglia cells and their phenotypes in the layers of the cerebral cortex, which are deceased from hemorrhagic stroke in the hyperacute phase, that is, when less than 24 h passed from the onset of the disease to death. We studied microglia phenotypes using digital photomicrographs of slides of specially stained brain samples. The results we obtain indicate a significant difference in the distribution of microglia between the group with stroke and the control group. The results of the study indicate that the microglia response occurs already in the early phase of hemorrhagic stroke.

## 1. Introduction

Stroke is the one of leading causes of death worldwide. About 25% of stroke patients die within the first month. Patients with hemorrhagic stroke, which accounts for 10–20% of all strokes, have a poor prognosis. The fatality rate for hemorrhagic stroke in the first month is approximately 50% [1]. In addition, most surviving patients suffer from complications that significantly reduce their quality of life.

Non-traumatic subarachnoid hemorrhage (SAH) accounts for approximately 9% of all strokes [2]. Ruptured aneurysms are responsible for approximately 80% of SAH cases. In 15% of SAH cases, the cause remains idiopathic. Approximately 30% of patients with SAH die within 30 days of the accident [3]. Most SAH survivors have long-term consequences: 60% have memory impairment, 75% have speech problems, and approximately 95% have cognitive and affective issues. Patients with SAH often suffer from a loss of hearing and smell. These sequelae and their combination significantly reduce quality of life [4,5].

Cellular and molecular biomarkers are important for understanding the pathophysiology of stroke [6,7]. Microglia are among the most promising biological markers.

Microglia are mesoderm-derived mononuclear phagocytes that migrate to the central nervous system during early embryonic development. After colonizing the brain, microglia proliferate and maintain the ability to self-renew throughout life, constituting approximately 5–12% of all cells in the central nervous system. Microglia have extremely diverse morphological characteristics and actively modify the shape of their processes and soma in response to a variety of stimuli. This broad morphological spectrum of microglial responses is thought to be closely related to their various functions both physiologically and in disease [8]. However, the morphological and physiological characteristics of microglia, as well as the structural and functional aspects of their interactions with neurons and other cells, are largely unknown [9,10,11,12].

Microglia have two-way communication with other brain cells and with peripheral immune cells [13]. Microglia cells detect damage and migrate in that direction, phagocytizing damaged cells, synapses, myelin remnants, and debris. Microglia can also damage neurons. At the same time, microglia remodel the intercellular matrix, form synapses, stimulate neurogenesis, assist oligodendrocytes in myelinating axons, and participate in antigen presentation [11,14,15,16,17,18,19,20,21,22,23,24]. Microglia do not respond in isolation but are part of complex cell networks that interact with such brain cells as neurons, astrocytes, cerebrovascular endothelial cells, and oligodendrocytes [22]. The microglial response begins with detecting signals from damaged neurons, after which microglial cells migrate to the damaged cells and change the phenotype depending on the state of the neuron. When a neuron releases signals like «eat me», «don’t eat me», and others, the microglial cells react accordingly [24,25].

Microglial research has advanced significantly over the past few decades. However, most studies still classify microglia into activated and inactive, “bad” and “good”. Given the multifunctionality of microglia and their role in the development, aging, and plasticity of the central nervous system, such a categorization of glia cannot be considered correct, as noted in the article by Paolicelli et al. [26]. In our work, we have followed the recommendations of this article.

Due to the large number of different morphological phenotypes and the very limited information on microglial changes in subarachnoid hemorrhage (SAH), we performed this study.

The aim: To identify the features of the distribution of various microglial morphological phenotypes in the layers of the cerebral cortex in the hyperacute phase of non-traumatic SAH.

## 2. Materials and Methods

The study was performed on human post-mortem brain material. We performed a retrospective analysis of forensic examination cases of deceased persons from February 2019 to September 2019. Cases that met the following criteria were included in the study: the interval from death to autopsy was no more than 24 h (time of death was determined based on documentation), a histological archive was available, and the immediate causes of death were non-traumatic subarachnoid hemorrhage (SAH group) and sudden cardiac death (SCD) or ischemic heart disease (IHD) (control group).

Cases with traumatic subarachnoid hemorrhage, malignancy, infectious disease, signs of traumatic injury, and a postmortem interval greater than 24 h were excluded from the study. Autopsies were performed at the Bureau of Forensic Medical Examination of the Department of Healthcare of the City of Moscow.

Organ samples for histological examination were taken during the forensic examination in accordance with the current legislation (Order 491N of the Ministry of Health of the Russian Federation dated 25 September 2023) to confirm and verify the cause and time of death. In accordance with the procedure for forensic examination, organ samples were routinely fixed in neutral 10% formalin solution for at least 24 h [27], followed by the standard processing and embedding of paraffin blocks. Histological sections were cut at a 4 µm thickness and stained with hematoxylin and eosin and by Nissl staining. Histological slides were examined with a Nikon Eclipse Ni-U microscope (Nikon Instruments Inc., Tokyo, Japan). Patient names or other unique identifiers were not used in this study. The study protocol was approved by the local ethics committee (protocol 2/21/5 dated 16 April 2021).

The identification of the microglial marker Iba-1 was performed by immunohistochemistry staining. Cases in which an autopsy was performed within 24 h of death were selected for immunohistochemical staining. The slides for immunohistochemistry (IHC) were deparaffinized in xylene and sequentially dehydrated in alcohols. High-temperature antigen retrieval was performed in citrate buffer pH 6 (Target Retrieval Solution, DAKO, Glostrup, Denmark). The sections were cooled, washed in three changes of distilled water, and incubated in 3% hydrogen peroxide solution to inhibit endogenous peroxidase activity. They were then washed with phosphate buffer (PBS IHC Wash Buffer + Tween, Cell Marque, Rocklin, CA, USA) (3 × 5 min). Blocking serum (Protein Block Serum, Abcam, Cambridge, UK) was used for 15 min to prevent the non-specific binding of primary or secondary antibodies to tissue proteins. The sections were then incubated for 1 h at 37 °C in a solution of rabbit polyclonal antibodies against the microglial marker protein Iba-1 (1:200 dilution in antibody diluent, Abcam, Cambridge, UK). The slides were washed with PBS (2 × 5 min). The reaction was visualized using the IHC HRP/DAB (ABC) detection kit for mice and rabbits (Abcam, Cambridge, UK). After washing in PBS, the sections were stained with hematoxylin. After washing in tap water, the sections were mounted under coverslips.

The number and phenotypes of Iba-1-stained microglial cells were evaluated on microphotographs obtained using a Nikon Eclipse Ni-U microscope, a digital camera, and NIS-Elements BR (Basic Research) software version 5.20 (Nikon, Tokyo, Japan): on panoramic slide images taken with a 20x lens, an area 2 mm wide and long from the pia mater to the border of the sixth layer and white matter was selected, and then this area was processed and evaluated. Because the area of the selected regions varied in different slides, the data were calculated for 1 mm^2^.

We used the following criteria to recognize the morphologic phenotypes of microglia. Cells that have equal lengths and widths visually were classified as round. Rod-type cells were those that were visually longer than they were wide. An amoeboid cell was defined as a microglial cell that has an amount of cytoplasm and contains protrusions and/or bends in its body.

Statistical analysis was performed using IBM SPSS Statistics 29.0. Nonparametric methods of statistical analysis, such as the Mann–Whitney U test, were used for intergroup comparisons. For within-group analysis, nonparametric methods such as Wilcoxon and Friedman tests with a posteriori analysis (Bonferroni correction for multiple comparisons) were used. Differences were considered significant at *p* < 0.05 (two-way criterion). Descriptive statistics were reported as the median and interquartile range.

## 3. Results

### 3.1. General Characteristics of SAH and Control Group

The total number of cases analyzed was 227. The number of cases with SAH was 28. During the selection, after excluding 178 cases with a post-mortem period longer than 24 h, 35 cases out of the remaining 49 cases were excluded due to non-compliance with the following criteria: absence of alcohol intoxication, absence of autopsy signs of infectious disease, and trauma. A total of 14 cases met the inclusion criteria.

Thus, we selected seven cases for IHC staining in the control group, including four males and three females, with a median age of 62 (57–72) years (in four cases, it was CDH, and in three cases, it was SCD).

The SAH group included seven cases, of which three were male and four were female, and the median age was 72 (60–78.5) years. Localization of SAH: in five cases, it was a rupture of the median cerebral artery, in one case, it was a rupture of the basilar artery, and in one case, it was a rupture of the communicating artery.

**Figure 1:**
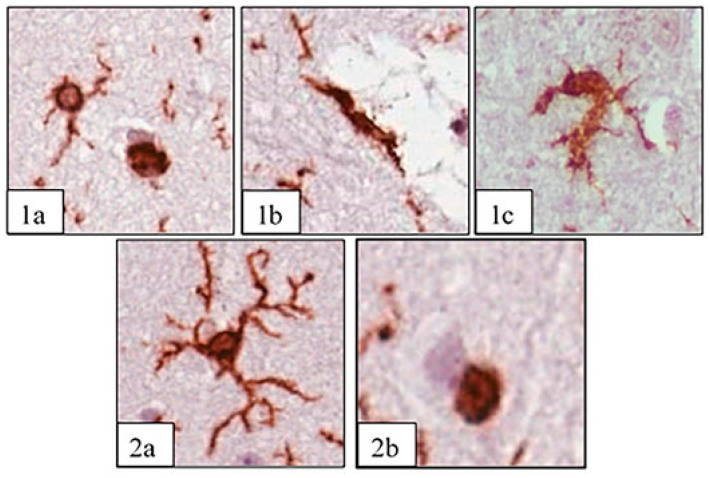
Phenotypes of microglia: round (**1a**), rod-type (**1b**), amoeboid (**1c**), ramified (**2a**), and non-ramified (**2b**). IHC staining Iba-1 (brown) and hematoxylin are shown as the contrast; the image was taken with a 20x lens. Example images were sourced from slides from the SAH group.

Based on the shape of the cell body and the presence of processes, the following microglial phenotypes were identified and analyzed: round rod-type, amoeboid, ramified, and non-ramified (Figure 1).

### 3.2. Morphological Characteristics of the Control Group

**Figure 2:**
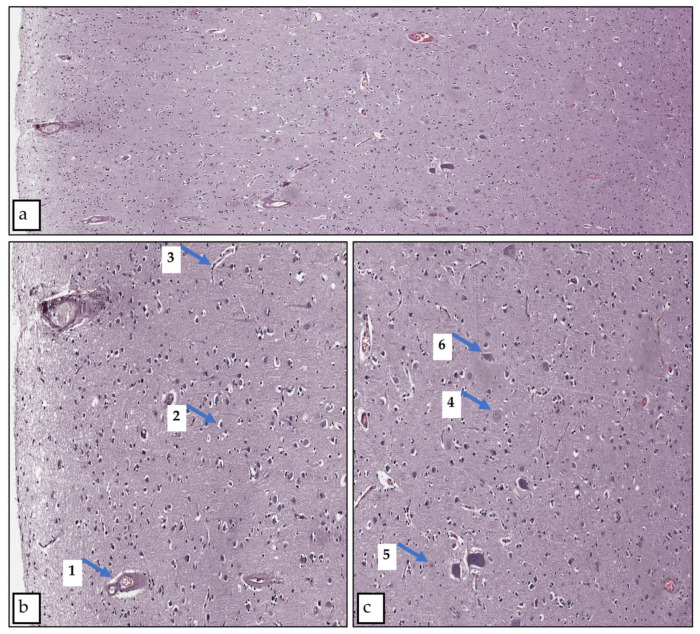
Cortical region of the control group slide Hematoxylin and eosin staining. Magnification 20x. (**a**) Overview photo of all layers, (**b**) layers one to three—close view, (**c**) layers four to six—close view. (1) Perivascular edema, (2) pericellular edema, (3) collapsed vessel, (4) swollen neuron, (5) evanescent cell, (6) dark neuron.

The pia mater was partially detached and thin, and its vessels were collapsed irregularly engorged. In the first layer of the cortex, the vessels were collapsed, and pericellular and perivascular edema of varying severity was seen. In the second to sixth layers of the cerebral cortex, the vessels were collapsed, and vessels with signs of hyalinosis were observed. There was edema in the perivascular and pericellular spaces and around glial elements of varying degrees. Dystrophic changes in neurons of varying severity were observed. Swollen neurons with a decentralized nucleus and nucleolus, such as evanescent cells, ghost cells, dark neurons, and cells with cytoplasm devoid of Nissl substance, were observed (Figure 2).

#### Immunohistochemical Study of Microglia in the Control Group

**Figure 3:**
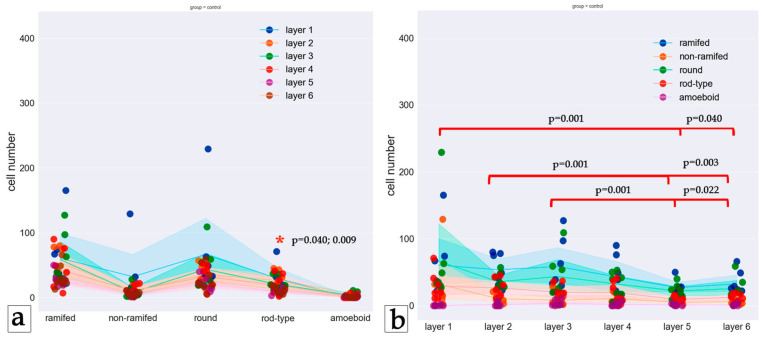
Distribution of microglia phenotypes in layers of the control group. (**a**) Phenotypes are shown in columns, and the layers are indicated by the color of the dots. (**b**) Layers are shown by columns, while phenotypes are represented by different colors of dots. Dots represent the number of cells, and lines represent the mean cell number according to the color scheme. Brackets point to significant differences in the number of cells between layers. Asterisks point to significant differences in the phenotype distribution in the layer. The Friedman test was used to analyze related samples.

**Figure 4:**
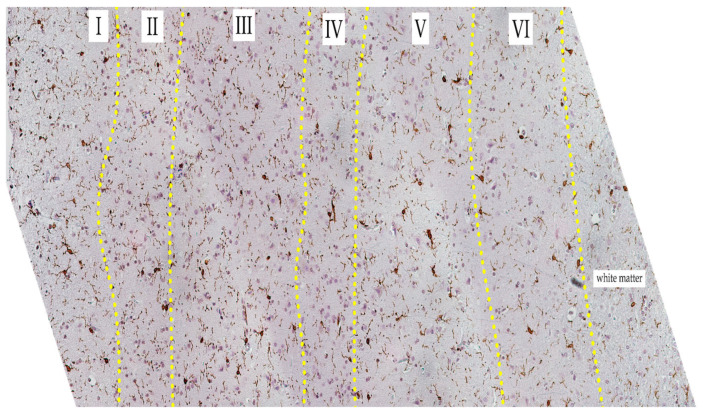
Cortical region of the control group slide. Iba-1 staining, hematoxylin contrast. Magnification 20x. The yellow dot-line indicates the border between layers. Layers are indicated in Roman numerals.

We obtained some significant variations in the distribution of microglia and their phenotypes in layers of the control group. The results indicate that the number of microglia in the fifth and sixth layers is lower than that in the first, second, and third layers (*p* ≤ 0.001; <0.001; <0.001; 0.040; 0.003; 0.022). Also, in the fifth layer, the number of rod-type microglia is lower than that in the first and second layers (*p* = 0.040; 0.009) (Figure 3 and Figure 4). More information presented in Supplementary Materials (Tables S1–S3).

### 3.3. Morphological Characteristics of the SAH Group

**Figure 5:**
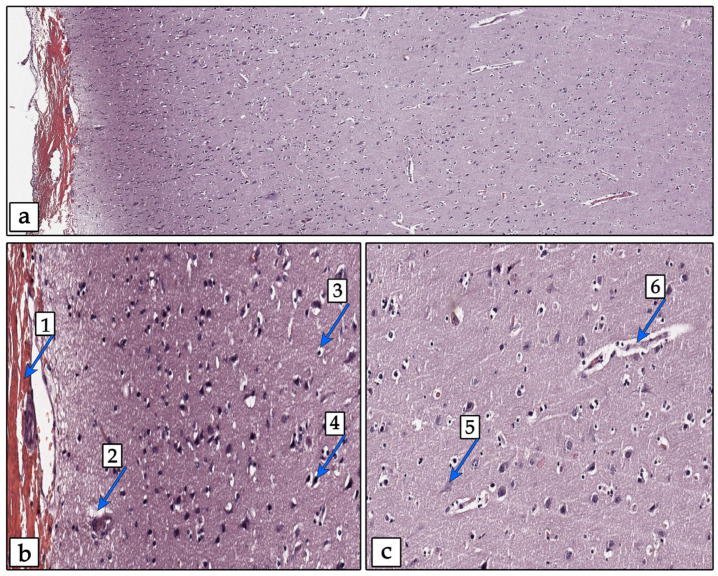
Cortical region of the SAH group slide. Hematoxylin and eosin staining. Magnification 20x. (**a**) Overview photo of all layers, (**b**) layers 1–3 close view, (**c**) layers 4–6 close view. (1) Hemorrhage, (2) perivascular edema, (3) pericellular edema, (4) dark neuron, (5) evanescent cell, (6) collapsed vessel.

The pia mater was irregularly thickened and partially or completely detached. There were hemorrhages both above and below the pia mater. The vessels of the pia mater had irregular blood filling, and the walls of some vessels were thickened due to plasmatic infiltration. The brain matter vessels had uneven blood filling and were partially collapsed. Perivascular, pericellular, and periglial edema ranged from moderate to severe. Dystrophic changes in neurons of varying severity and neuronophagia were observed (Figure 5).

#### Immunohistochemistry of Microglia in the SAH Group

**Figure 6:**
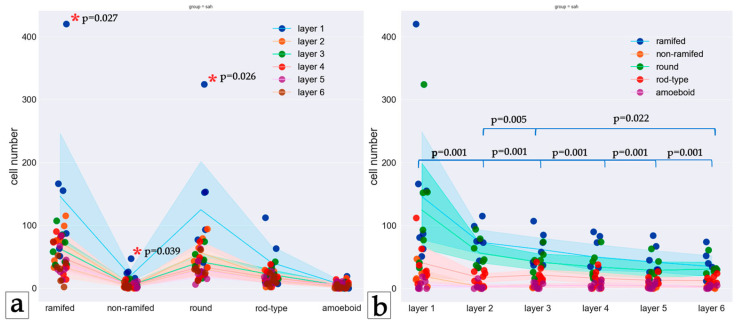
Distribution of microglia phenotypes in layers of the SAH group. (**a**) Phenotypes are shown in columns, and the layers are indicated by the color of the dots. (**b**) Layers are shown by columns, while phenotypes are represented by different colors of dots. Dots represent the number of cells, and lines represent the mean cell number according to the color scheme. The Friedman test was used to analyze related samples.

**Figure 7:**
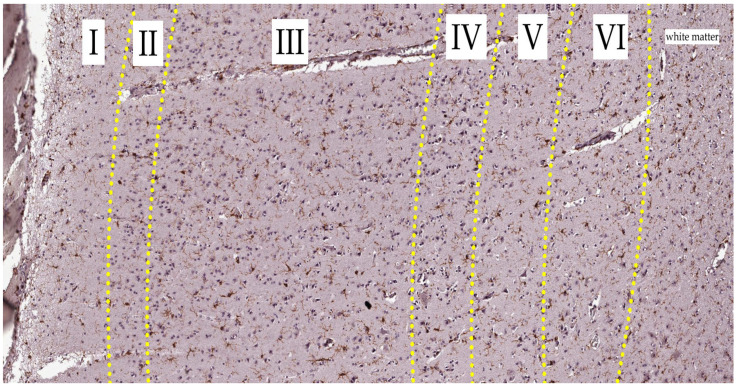
Cortical region of the SAH group slide. Iba-1 staining, hematoxylin contrast. Magnification 20x. Yellow dot-line indicates the border between layers. Layers indicated in Roman numerals.

In the SAH group, we obtained the following significant results. In the first layer, the number of microglia was higher than that in the second to sixth layers (*p* = 0.001), in the sixth layer, the number of cells was lower than that in the second and third layers (*p* = 0.005; 0.022), and the first layer had more ramified, non-ramified, and round cells than the fifth and sixth layers (*p* = 0.027; 0.039; 0.026) (Figure 6 and Figure 7). More information is presented in Supplementary Materials (Tables S4–S6).

### 3.4. Results of the Comparative Analysis of Cortical Microglia in the SAH Group and Control Group

**Figure 8:**
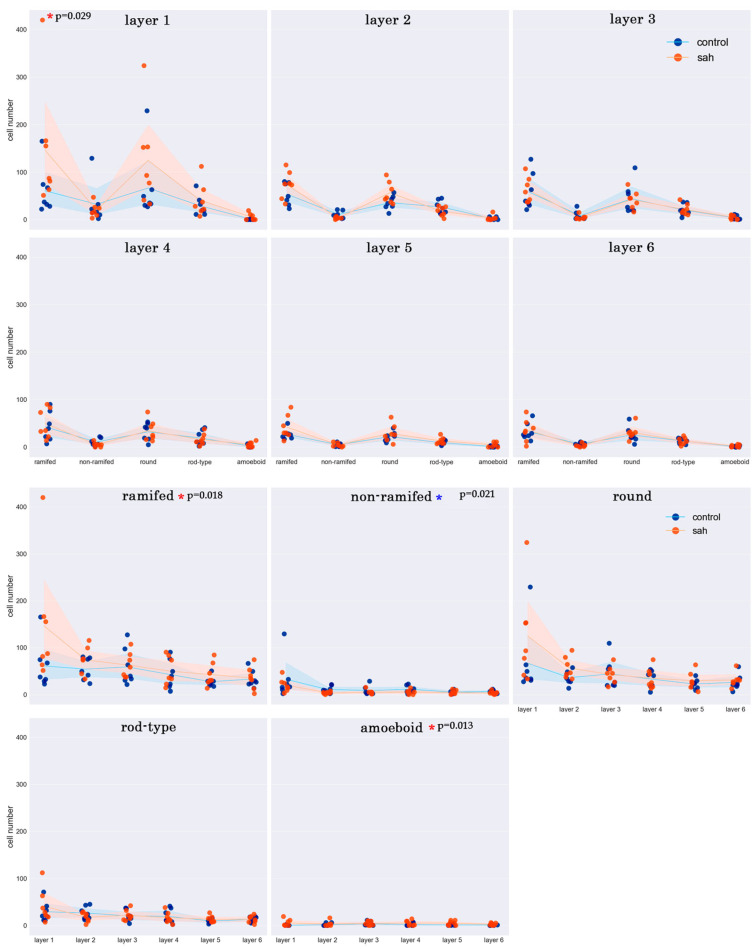
Distribution of microglia cells by layers and phenotypes in the control and SAH groups. Dots represent the number of cells, and lines represent the mean cell number according to the color scheme.

The following significant results were obtained when comparing between groups: in layer one, the total number of cells was higher in the SAH group (35 (14–85)) than in the control group (21 (8–42)) (*p* = 0.029). Phenotype comparisons revealed that ramified and amoeboid cells were more numerous in the SAH group than in the control group (*p* = 0.029; 0.013). Non-ramified cells were less numerous in the SAH group than in the control group (Figure 8). More information presented in Supplementary Materials (Table S7; Figure S1).

## 4. Discussion

The results of the study revealed quantitative and qualitative rearrangements of morphological phenotypes of microglia in the cerebral cortex in subarachnoid hemorrhage. This raises questions that require clarification and justification.

First, is there quantitative change in the microglial content in the human cerebral cortex during hyperacute SAH? Our results show that the number of microglia varies: in the SAH group, there were more cells in layer one than in the control group (*p* = 0.029). This coincides with the data of experimental studies [28,29,30,31].

Second, were there significant differences in the content of microglia in the SAH group in different layers of the cerebral cortex? The number of microglia in layer 1 was higher than that in all other layers, and the number of microglia in layer six was lower than that in layers two and three. These data are consistent with data from experimental studies that showed an increase in the number of microglia around the lesion center [28,29,30]. In the control group, the number of microglia in layers five and six was significantly lower than that in layers one, two and three. It is noteworthy that both groups showed a lower number of microglia in layers five and six compared to layers one, two, and three.

The literature reports that microglia are characterized by interregional heterogeneity. Microglia specific for the cortex, hippocampus, cerebellum, subventricular zone, and thalamus have been identified in ischemic brain damage [32,33].

Vascular-associated microglia (VAM) and perivascular macrophages (PVM) can be distinguished by their different morphologies, signatures, and microscopic locations. As a key component of the neuroglial vascular unit (NVU), they play important roles in neurovascular development and pathogenesis, including phagocytosis, angiogenesis, and vascular injury/protection [34,35].

We investigated the distribution of microglia in the hyperacute phase of SAH. There are two phases in early SAH: the period of early brain injury and the period of delayed brain ischemia. The period of early brain injury begins from the moment of hemorrhage, which leads to increased intracranial pressure, impaired cerebral perfusion, and global ischemia. This period continues for 72 h from the onset of SAH. During the first phase, microglia polarize predominantly in the phagocytic type. In the second phase, after 72 h, the balance of microglia polarization shifts towards the reparation type [1,4,5,36,37].

In our study, we can speak of a period of early brain damage, as death occurred rapidly due to blood bursting into the ventricles or brain displacement. The second phase is characterized by the development of cerebral edema, which can be classified into three types: vasogenic, cytotoxic, and mixed. Vasogenic cerebral edema is the most common, while cytotoxic edema is caused by increased permeability as a result of cell membrane damage. Erythrocyte lysis products, which impregnate surrounding tissues during hemorrhage, also play an important role. In this way, secondary damage occurs in SAH [5,36,38,39,40].

Experimentally, microglia have been shown to be preconditioned, resulting in the better recovery of neurological function [41].

Notably, there are sporadic reports in the available literature on the study of microglia in human hemorrhagic stroke [3,42].

The third question is whether differences in the distribution of morphological phenotypes of microglial cells in different layers of the cerebral cortex were detected both within each of the two groups studied and between groups. Microglia are known to be very plastic and change their phenotype depending on the microenvironment [43,44,45,46,47]. There are data linking processes’ length to the functional status of microglia. The stimulation of processes elongation has been shown to reduce the secretion of cytokines that promote change and restore the ability of microglia to scan the environment [48,49].

We categorized microglia into the following morphological phenotypes: rod-type, round, non-ramified, ramified, and amoeboid. Many morphological phenotypes of microglia have been identified in published studies using morphometric analysis methods, including those based on machine learning, such as homeostatic, activated, reactive, hypertrophic, dystrophic, non-ramified, branched, hyperbranched, spiny, bushy, inflamed, rod-type, amoeboid, large amoeboid, Hitter cells, “fried egg”, bipolar, honeycomb-shaped, and jellyfish [6,44,47,50,51,52,53,54,55,56,57]. To date, there is no uniform classification of microglial morphological phenotypes, which makes it difficult to compare the results of different studies.

When comparing phenotypes in the control group, the differences in the number of rod-type microglial cells in layer five were significant: there were fewer of them than in layers one and two. Pairwise comparisons between cell types within layers showed that in all layers, there were significantly fewer amoeboid cells than round cells, and in layers two, three, five, and six, the number of microglial cells with processes was significantly lower than the number of microglial cells without processes. In layer two, there were significantly fewer amoeboid cells than rod-type microglial cells. Thus, our data confirm existing reports that ramified microglia normally predominate [30,48].

There were significant differences in the distribution of phenotypes in the cortical layers in the SAH group: the number of ramified, non-ramified, round, and rod-type microglial cells were greater in layer one than in layer five; ramified and non-ramified microglial cells were more numerous in layer one than in layer six; there were more round microglial cells in layer two than in layer five. Layer one had more microglia, both non-ramified and ramified, than layer six. Pairwise comparisons of cell phenotype distributions within layers revealed that amoeboid and non-ramified cells were less common than round and ramified cells in all layers. Layers three and six contained fewer amoeboid cells than rod-type cells. In all layers, ramified cells predominated over rod-type cells. In layer two, there were more round cells than rod-type ones. Layers three and six contained more rod-type cells than amoeboid or non-ramified cells. The literature shows that ramified microglia cells predominate in the first three days following SAH. Later, the balance shifts toward amoeboid cells [30,31].

When phenotypes were compared between groups, significant differences were found: the SAH group had more ramified and amoeboid cells than the control group. There were fewer non-ramified cells in the SAH group compared to the control. Satellite cells were more numerous in the SAH group than in the control group, which is consistent with previous findings.

Our study has some limitations, mainly due to its retrospective and forensic nature, such as a small sample size, a lack of information on previous treatment measures, and the use of a single microglial marker (Iba-1) for IHC staining. However, the study provided results indicating changes in the morphological and quantitative composition of microglia in SAH. The findings are scientifically novel and serve as a basis for future research into the distribution of microglial morphological phenotypes in the layers of the cerebral cortex in various diseases intended to identify features and potential therapeutic interventions. Given the rapid response of microglia to subarachnoid hemorrhage, further research is needed to assess the effect of the duration of the dying period from SAH to death on quantitative and qualitative morphological changes in microglia.

For future studies, we plan to use additional markers of microglia. Each marker is a protein with its own function. IBA-1 is an actin-binding protein expressed in macrophages that is required for microglial membrane ruffling [58,59]. The use of other microglia markers in further studies will allow for a more detailed understanding of not only the morphological but also the functional changes in microglia.

## 5. Conclusions

Compared with controls, the SAH group had a higher number of cells in the first layer (*p* = 0.029), which was in direct contact with the hemorrhage, and it also had a predominance of ramified and amoeboid phenotypes (*p* = 0.029; 0.013). Thus, the statistically significant difference indicates both quantitative and phenotypic changes in microglia in early SAH in the human cortex.

## Data Availability

Data are contained within the article and Supplementary Materials.

## Supplementary Materials

The following supporting information can be downloaded at: https://susy.mdpi.com/user/manuscripts/displayFile/99195e93cb869d483a08bfa4cf4c0512/supplementary, Figure S1: Heat map of the distribution of morphological phenotypes in cerebral cortical layers in groups control and SAH. Color indicates the number of microglial cells according to the color scale; Table S1: Significant results of comparisons of microglial cell counts by layer in the control group; Table S2: Significant results of comparisons of microglial phenotypes in layers; Table S3: Significant results of pairwise comparisons of microglial cell phenotypes in layers in the control group; Table S4: Significant results of comparisons of the number of microglial cells in different layers of the cortex in the SAH group; Table S5: Significant results of comparisons of the number of microglial cells in different layers of the cortex in the SAH group; Table S6: Significant results of pairwise comparisons of microglial cell phenotypes in the layers; Table S7: Significant results of comparisons of microglial cell phenotype distributions between groups.
